# Nanomaterial-mediated antibiotic delivery: a novel strategy for osteomyelitis therapy

**DOI:** 10.3389/fbioe.2025.1671151

**Published:** 2025-09-30

**Authors:** Shuang Cheng, Xiao-Hui Meng, Zhi Li, Hai-Hui Han, Ya-Feng Zhang

**Affiliations:** ^1^ Nanjing University of Chinese Medicine, Nanjing, China; ^2^ Jiangsu CM Clinical Medical Innovation Center of Degenerative Bone and Joint Disease, Wuxi Affiliated Hospital of Nanjing University of Chinese Medicine, Wuxi, China; ^3^ Orthopedics Department, Shanghai Minhang Hospital of Integrated Traditional Chinese and Western Medicine, Shanghai, China

**Keywords:** osteomyelitis, nanomaterials, antibiotic delivery, targeted therapy, bone regeneration

## Abstract

Osteomyelitis is an inflammatory bone disease caused by bacterial infection, often leading to bone destruction and functional impairment. Traditional treatments face significant limitations, including substantial surgical trauma, low drug delivery efficiency, and a high risk of recurrence. Nanomaterial-mediated antibiotic delivery has emerged as an innovative strategy, enabling localized, targeted and controlled antibiotic release. Representative platforms include nanohydroxyapatite (nHA), mesoporous bioactive glass (MBG), poly (lactic-co-glycolic acid) (PLGA), metal–organic frameworks (MOFs), silver nanoparticles (AgNPs), and multifunctional hybrid composites. This approach can enhance therapeutic efficacy, reduces systemic side effects, and promotes bone regeneration. This review summarizes the pathogenesis and therapeutic challenges of osteomyelitis, explores the construction and delivery mechanisms of nanocarriers, and discusses recent advances from *in vitro* studies to animal models and clinical research. Current evidence indicates that nanocarrier-based drug delivery systems can effectively inhibit bacterial growth, modulate inflammatory responses, and facilitate bone regeneration. However, their large-scale clinical application remains limited by concerns regarding safety, manufacturing complexity, regulatory standardization, and cost. Future directions include the development of intelligent nanocarriers, integration with multimodal therapeutic strategies (e.g., photothermal, immunomodulatory, and stem cell-assisted therapies), establishment of standardized multi-tier toxicity evaluation frameworks, and progression toward large-animal validation and early phase clinical trials, which are expected to drive further progress and provide more effective and safer treatment options for osteomyelitis.

## 1 Introduction

Osteomyelitis is a bone infection resulting from bacterial invasion of the bone marrow, cortical bone, and periosteum, often accompanied by sequestrum formation and it frequently leads to bone destruction and necrosis, as well as functional impairment, and may ultimately result in permanent disability in affected patients. When the infection occurs in proximity to a joint, it can cause joint contracture, stiffness, and restricted mobility (Lew and Waldvogel, 2004; Kavanagh et al., 2018). Recent population-based data show an overall incidence of 21.8 per 100,000 persons, higher in males; approximately 13 per 100,000 in children versus 90 per 100,000 in adults (Kremers et al., 2015). In diabetes, 19%–34% of patients develop a lifetime diabetic foot ulcer and a substantial proportion progress to osteomyelitis (McDermott et al., 2023). Each complex episode imposes considerable clinical and economic burden: the median hospital stay is about 22 days, and the median inpatient cost for post-traumatic osteomyelitis (USD 10,504) is 4.8-fold that of non-traumatic cases (USD 2,189) (Jiang et al., 2020). These epidemiological and economic indicators underscore the need for more effective, cost-efficient strategies.

The primary treatment for osteomyelitis involves surgical debridement combined with antibiotic therapy. However, conventional therapeutic approaches present several limitations. Surgical intervention is highly invasive, requires a prolonged recovery period, and may result in bone defects or disease recurrence (Zhang H. et al., 2025). Systemic antibiotic therapy frequently fails to achieve and maintain effective drug concentrations at the infection site due to poor vascularization, thereby leading to suboptimal therapeutic outcomes (Abdulrehman et al., 2024). Although local administration can deliver higher drug concentrations to the infected area, commonly used carriers (such as bone cement or calcium sulfate) typically involve simple physical mixing with antibiotics. This approach often results in uneven drug distribution and burst release, thereby making it challenging to achieve sustained and controlled antibiotic delivery (Štícha et al., 2023; Birk et al., 2020).

Nanomaterials, defined as materials with structural dimensions ranging from 1 to 100 nm, exhibit a high surface-to-volume ratio, advantageous mechanical properties, and reactive surface chemistry (Hammond, 2011; Zapata et al., 2022; Eivazzadeh-Keihan et al., 2024). These features enhance their capacity for drug adsorption and controlled release, thereby demonstrating significant potential for the treatment of osteomyelitis. As an emerging therapeutic strategy, nanomaterial-mediated antibiotic delivery offers new prospects by enabling localized drug delivery, minimizing systemic side effects, and providing controlled and sustained antibiotic release. Moreover, this approach reduces toxicity to surrounding tissues and promotes bone regeneration (Geurts et al., 2021; Liu et al., 2022). Therefore, comprehensive research into nanomaterial-mediated antibiotic delivery is essential for improving therapeutic outcomes in osteomyelitis.

Although some excellent reviews summarize multifunctional strategies for nanocarriers and bone infection (Geurts et al., 2021; Zapata et al., 2022), this review shows differences from current research progress. First, we highlight recent advances in intelligent (stimulus-responsive) nanocarriers and multifunctional composite scaffolds that have not been previously covered. Second, we propose a cross-mapping framework that links material types to therapeutic functions (infection control, immunomodulation, and bone regeneration) to reduce redundancy and facilitate translation-oriented vector selection. Finally, we have dedicated discussions on clinical translation, regulatory considerations, and industrial scalability, topics that are critical to moving nanomedicines from the lab to the bedside but were not fully addressed in earlier reviews.

## 2 Overview of osteomyelitis

Osteomyelitis is an inflammatory disease of bone tissue caused by infection with bacteria, fungi, or other pathogens (Yang et al., 2024). The condition involves not only the bone marrow but also the cortical bone and periosteum. According to etiology, clinical course, and route of infection, osteomyelitis can be categorized into several types, among which hematogenous osteomyelitis, post-traumatic osteomyelitis, and contiguous spread osteomyelitis are the most prevalent (Ziran, 2007). Pediatric and adult osteomyelitis differ in vascular anatomy, sites, pathogens and presentation: children typically develop acute hematogenous infection in long-bone metaphyses (rich but slow metaphyseal flow across an open growth plate), with abrupt fever and pain; adults more often have vertebral, post-traumatic or diabetic foot involvement, insidious onset, frequent biofilm and polymicrobial (including Gram-negative) infection, and higher chronicity and comorbidity burden (e.g., diabetes, peripheral vascular disease) (Birt et al., 2017).

The pathogenesis of osteomyelitis is highly complex, involving multiple interconnected processes such as microbial infection, inflammatory responses, bone destruction, and host immune reactions (Masters et al., 2019). *S. aureus* is the most prevalent bacterial pathogen responsible for osteomyelitis (Kavanagh et al., 2018; Urish and Cassat, 2020; Chen Y. et al., 2023). When bacteria invade bone tissue via the bloodstream, trauma, or contiguous spread from adjacent tissues, they initially colonize the bone marrow cavity and proliferate extensively. The toxins and enzymes released by these bacteria subsequently destroy surrounding osteoblasts and the bone marrow stroma, thereby triggering inflammatory responses (Granata et al., 2022; Johnson et al., 2023). Inflammatory cells, including neutrophils and macrophages, rapidly accumulate at the site of infection and secrete a variety of proinflammatory mediators—such as tumor necrosis factor-α (TNF-α), interleukin-1 (IL-1), and interleukin-6 (IL-6)—which amplify the inflammatory response and resulting in local hyperemia, edema, and pain. As inflammation progresses, bone destruction is further exacerbated (Kang et al., 2018). With persistence, an immune-evasive milieu emerges. Biofilm matrix (PNAG, extracellular DNA) and agr down-regulation reduce PAMP exposure, while small-colony variants shift metabolism and dampen ROS triggers. *S. aureus* engages TLR2-PI3K/Akt-STAT3 and MAPK pathways to elevate IL-10, and induces TGF-β, skewing macrophages toward an M2/repair phenotype (Arg1, SOCS3, reduced iNOS) (Branquist and Kielian, 2025; Rasquel-Oliveira et al., 2025). These cytokines expand FOXP3+ regulatory T cells and upregulate PD-1/PD-L1, restraining Th1/Th17 effector activity. Concomitantly, MDSCs accumulate, secreting IL-10 and depleting L-arginine, further impairing neutrophil and T-cell function. Collectively, spatial sequestration (deep foci, biofilm) and these immunosuppressive circuits reduce antibiotic and immune penetration, rendering eradication difficult (Lindsay et al., 2021).

**FIGURE 1:**
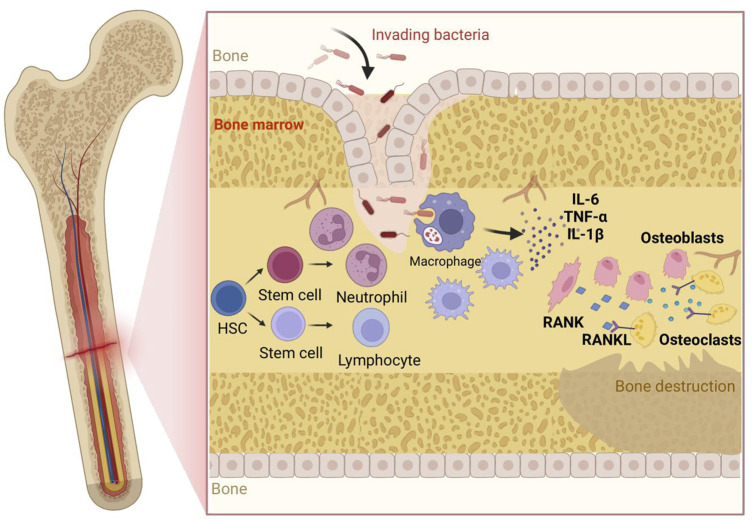
Pathogenesis of osteomyelitis:Bacteria invade bone/marrow, adhere and initiate (pre-)biofilm formation, driving rapid neutrophil influx; macrophages polarize to M1 releasing IL-1β, IL-6, TNF-α, amplifying inflammation and—together with lymphocyte/stromal cues—upregulating RANKL, which engages RANK on hematopoietic precursors to generate active osteoclasts; heightened resorption plus impaired osteoblast function yields bone destruction; persistent inflammatory/biofilm signals suppress MSC osteogenesis and prolong immune activation, worsening loss. Abbreviations: IL-6, Interleukin-6; TNF-α, Tumor Necrosis Factor-α; IL-1β, Interleukin-1β; RANK, Receptor Activator of Nuclear Factor κB; RANKL, Receptor Activator of Nuclear Factor κB Ligand; HSC, Hematopoietic Stem Cell; OC, Osteoclast; OB, Osteoblast; MSC, Mesenchymal Stem Cell; BM, Bone Marrow; Neu, Neutrophil; Mac (Mφ), Macrophage; Lym, Lymphocyte; EPS (optional), Extracellular Polymeric Substance; ROS (optional), Reactive Oxygen Species.

Current treatment strategies for osteomyelitis mainly comprise surgical debridement, systemic antibiotic therapy, and local drug delivery (Masters et al., 2022), as illustrated in Figure 1. Nevertheless, each of these modalities is associated with significant clinical challenges. Surgical debridement is a cornerstone in managing osteomyelitis, aiming to remove necrotic tissue, sequestra, and foreign bodies to reduce bacterial load and establish a favorable environment for subsequent therapies (Zhou et al., 2021). However, this procedure is invasive and may inadvertently damage adjacent healthy bone and soft tissue, thereby adversely affecting bone healing and limb function. Systemic antibiotic therapy, delivered orally or intravenously, remains fundamental to treatment. However, owing to poor vascularization at infected bone sites, achieving and maintaining effective drug concentrations is often challenging, resulting in suboptimal therapeutic outcomes (Mills et al., 2018). Moreover, the widespread use of antibiotics has led to the emergence of resistant bacterial strains, further diminishing the efficacy of conventional antimicrobial agents. Local antibiotic delivery aims to achieve high drug concentrations at the infection site while minimizing systemic side effects. Although elevated local antibiotic concentrations can effectively control infection, they may also exert cytotoxic effects on surrounding tissues, interfere with new bone formation, and ultimately impair bone repair and regeneration (Mulazzi et al., 2021; Mills et al., 2024).

## 3 Strategies for nanomaterial mediated antibiotics

### 3.1 Types of nanocarriers

**FIGURE 2:**
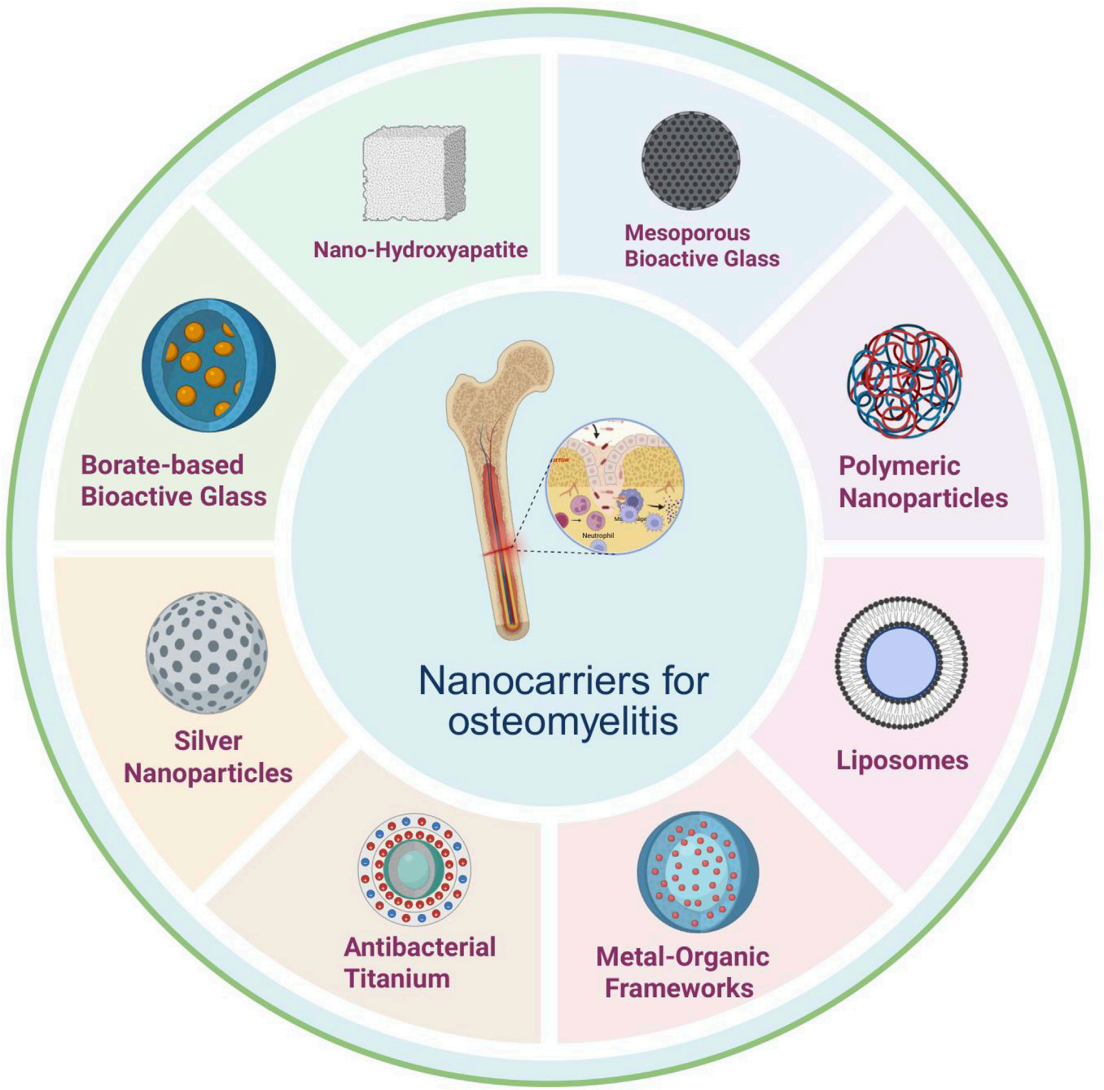
Nanodelivery materials in osteomyelitis: principal classes include nano-hydroxyapatite, silver nanoparticles, metal–organic frameworks, liposomes, antibacterial titanium, borate-based bioactive glass, mesoporous bioactive glass, and polymeric nanoparticles, offering combinable advantages in localized high loading and controlled release, antimicrobial/biofilm disruption, osteoconduction/osteogenesis support, structural reinforcement, and surface functionalization capacity. Abbreviations: nHA, nano-hydroxyapatite; AgNPs, silver nanoparticles; MOFs, metal-organic frameworks; Lipo, liposomes; Ti (antibacterial Ti), antibacterial titanium (surface-modified titanium); BBG (or B-BG), borate-based bioactive glass; MBG, mesoporous bioactive glass; PNPs, polymeric nanoparticles. (Optional if used elsewhere: EPS, extracellular polymeric substance; ROS, reactive oxygen species).

Types of Nanomaterials and Their Applications in Osteomyelitis Therapy. In the context of osteomyelitis treatment, a variety of nanomaterials have emerged as promising antibiotic carriers owing to their unique physicochemical properties and biological activities, thereby offering new opportunities to enhance therapeutic efficacy. Common nanomaterials are shown in Figure 2.

Nano-hydroxyapatite (nHA) is a nanomaterial that closely resembles the inorganic component of human bone in chemical composition. It exhibits excellent biocompatibility and osteoconductivity, promoting the adhesion, proliferation, and differentiation of osteoblasts, thereby providing an ideal scaffold for bone repair and regeneration (Hassan et al., 2016). Studies have demonstrated that vancomycin-loaded nHA, when combined with polylactic acid, is effective in the treatment of chronic osteomyelitis. This approach not only eradicates bacteria but also promotes the repair of bone defects and improves treatment success rates (Lv et al., 2022). To avoid rapid degradation of growth factors and minimize antibiotic-related side effects, a dual-controlled release microsphere scaffold system based on nHA has been developed, which further facilitates bone regeneration (Zhan et al., 2024).

Mesoporous bioactive glass (MBG) is a novel bioactive material distinguished by a highly ordered mesoporous structure and large specific surface area, features that enable efficient antibiotic loading (Guduric et al., 2021). Upon degradation *in vivo*, MBG releases bioactive ions that significantly stimulate bone regeneration by promoting the proliferation and differentiation of osteoblasts while simultaneously inhibiting osteoclast activity (Jia et al., 2017; Zhao et al., 2023). Studies have demonstrated that antibiotic-loaded MBG can effectively suppress infection, promote bone tissue regeneration, and attenuate inflammation in the treatment of osteomyelitis (Rahaman et al., 2014; Aurégan and Bégué, 2015; Kojima et al., 2021). Among the various types of MBG, S53P4 is the most widely utilized and has achieved excellent clinical outcomes in multiple studies (Romanò et al., 2014; Ferrando et al., 2017), exhibiting both effective local infection control and enhanced bone regeneration. In a rat tibial defect osteomyelitis model, vancomycin-loaded MBG granules (S53P4 base) reduced *S. aureus* counts by approximately 2 log compared with systemic therapy alone and enhanced new bone volume (Zhang P. et al., 2023). Clinically, S53P4 bioactive glass achieved infection eradication rates of 85%–92% in chronic osteomyelitis cohorts after debridement, comparable or superior to antibiotic PMMA beads while supporting bone regeneration (Lindfors et al., 2010).

Polymeric nanoparticles, synthesized from either natural or synthetic polymers, offer considerable design flexibility and a variety of drug loading mechanisms. These nanoparticles can incorporate antibiotics through physical encapsulation or chemical conjugation, thereby enabling stable drug loading and sustained release (Chopra et al., 2021). For example, poly (lactic-co-glycolic acid) (PLGA) copolymer nanoparticles exhibit excellent biodegradability and controlled drug release properties, allowing for slow degradation and sustained antibiotic release *in vivo*. This helps maintain therapeutic drug concentrations at the infection site and reduces the frequency of drug administration (Shakya et al., 2023; Lehner et al., 2025). Vancomycin-loaded aragonite nanoparticles (VANP) have also been investigated for osteomyelitis treatment (Saidykhan et al., 2016). These studies validate VANP as a promising candidate for the development of topical antibiotic delivery systems against osteomyelitis, demonstrating optimal antimicrobial efficacy, favorable bone resorption characteristics, and good biocompatibility.

Liposomes are vesicular structures composed of phospholipid bilayers, exhibiting excellent biocompatibility and biodegradability. They are capable of encapsulating both hydrophilic and hydrophobic antibiotics, thereby protecting the drugs from environmental degradation and enhancing its stability (Wang et al., 2019; Dymek and Sikora, 2022). The surfaces of liposomes can be functionalized to achieve active targeting, thereby increasing antibiotic accumulation at infection sites. Furthermore, liposomes can enter cells via membrane fusion or endocytosis, facilitating intracellular drug delivery and enhancing the eradication of intracellular pathogens (Zhang et al., 2018; Jose et al., 2019; Chen et al., 2021).

Metal-organic frameworks (MOFs) are porous materials through the self-assembly of metal ions or clusters with organic ligands. They are characterized by high tunability, large porosity, and high specific surface area, providing an ideal platform for efficient loading and controlled release of antibiotics (Qin et al., 2023). ZIF-8, a representative MOF, can encapsulate various antibiotics through coordination or physical adsorption (Zhu et al., 2024). In the treatment of osteomyelitis, antibiotic-loaded ZIF-8 can be slowly degraded in the micro-acidic environment of the infected site, enabling continuous antibiotic release for precise bacterial eradication (Shao et al., 2024). Additionally, MOF materials can be further enhanced by functional modification of organic ligands to confer additional functions, such as immunomodulation, thereby further improving therapeutic effect (Ge et al., 2022). In a murine *S. aureus* femoral osteomyelitis model, vancomycin@ZIF-8 exhibited pH-triggered release, lowering bacterial burden and preserving trabecular architecture versus free vancomycin (Liang et al., 2025).

In addition to the aforementioned nanocarrier systems, several specialized materials and technologies have also demonstrated significant therapeutic potential. Antibacterial titanium materials, for example, can be functionalized with silver ions via surface modification techniques, enabling the slow release of silver ions to inhibit bacterial growth (Macwan et al., 2017). These materials can exert antibacterial effects independently or be combined with antibiotics to achieve synergistic antibacterial activity, thereby significantly reducing the incidence of implant-associated osteomyelitis. Additionally, silver nanoparticles, exhibit strong inhibitory effects against pathogens. *In vivo* biodistribution and safety: Systemically or locally released AgNPs display size- and surface chemistry-dependent accumulation primarily in liver and spleen, with secondary deposition in lung and bone marrow (Jiang et al., 2020; Kumar et al., 2023). Partial oxidative dissolution liberates Ag+, which binds thiol-rich proteins and is progressively transformed into Ag2S or Ag2Se, decreasing acute ionic reactivity (Zhang et al., 2022). Therapeutic local concentrations generally spare osteoblast viability, whereas higher or repeated exposures induce mitochondrial ROS, inflammatory mediator upregulation, and delayed bone remodeling (Castiglioni et al., 2017). Monitoring cumulative silver burden and oxidative stress biomarkers is advisable for prolonged or repeated applications. However, when used alone, their therapeutic efficacy is comparable to that of classical antibiotics combined with local surgical debridement, suggesting that their synergistic effects with other therapeutic modalities should be comprehensively considered in clinical applications (Ip et al., 2022). AgNP-coated titanium or chitosan-stabilized AgNP composites curtailed *S. aureus* biofilm formation and reduced bacterial load in rabbit or rat long-bone infection models while maintaining acceptable local tissue responses (Chen X. et al., 2023). Borate glass, as a novel bioactive material, is capable of releasing ions to support proliferation, differentiation, osteogenic gene expression, and protein secretion of human bone marrow mesenchymal stem cells (Kędziora et al., 2020). In a rabbit infected bone defect model, antibiotic-loaded borate bioactive glass scaffolds facilitated rapid ionic (B, Ca) release, suppressed *S. aureus* colonization, and promoted vascularized new bone formation relative to inert carriers (Zhang et al., 2010; Jia et al., 2015). The ongoing development and application of these materials and technologies provide more options and possibilities for the treatment of osteomyelitis.

### 3.2 Construction of nano-antibiotic carriers

The construction of efficient nano-antibiotic carrier systems is a crucial step toward achieving nanomaterial-mediated antibiotic delivery. This process involves a variety of physical and chemical methods, as summarized in Table 1. The goal is to achieve stable binding of antibiotics to nanomaterials while ensuring that the carrier systems demonstrate optimal performance.

**TABLE 1:** The construction methods of nano-antibiotic carrier systems.

Construction method	Action principle	Advantages	Disadvantages	Reference
Physical Adsorption	Utilizes physical forces such as van der Waals forces and electrostatic interactions between the surface of nanomaterials and antibiotic molecules for adsorption	Simple operation, minimal impact on the structure and activity of drugs	Low drug loading capacity; weak binding force between drugs and carriers, prone to drug leakage	Wu J. et al. (2022)
Entrapment Method	Encapsulates antibiotics inside nanomaterials (such as polymer nanoparticles and liposomes)	Effectively protects drugs, improves stability, and enables higher drug loading	Complex preparation process, which may affect the drug release rate	Park et al. (2021)
Chemical Conjugation	Introduces specific functional groups on the surface of nanomaterials through chemical reactions, and then forms covalent bonds with corresponding functional groups on antibiotic molecules to achieve stable connection	Firm chemical bonding between drugs and carriers, preventing leakage and improving drug loading stability	Requires chemical modification, may involve complex reaction steps, and has high operational requirements	Sharifuzzaman et al. (2019)

Physical adsorption is one of the simplest approaches, in which antibiotics are attached to the surface of nanomaterials through non-covalent interactions such as van der Waals forces and electrostatic attraction. For instance, mesoporous silica nanoparticles possess abundant surface silanol groups (Saeting et al., 2022), which confer hydrophilicity and surface charge, enabling the adsorption of oppositely charged antibiotic molecules via electrostatic interactions. Surface functionalization can markedly enhance adsorption: acid-induced carboxylation of carbon nanotubes adds-COOH groups to bind cationic aminoglycosides; amination of silica with APTES introduces-NH3+ sites favoring anionic β-lactams; PEI modification of graphene oxide increases positive charge and available area, often raising loading 2-3 fold (Alosime, 2023). In one study, silica nanoparticles were used to adsorb interleukin-13 (IL-13), which successfully directed macrophage polarization both *in vitro* and *in vivo*, and alleviated experimental autoimmune encephalomyelitis (Zhang H. et al., 2023). Physical adsorption is easy to implement and minimally affects the structure and bioactivity of the drug. However, it typically results in relatively low drug loading and weak interactions between the drug and carrier, thereby increasing the risk of drug leakage during transport.

Encapsulation, or entrapment, is a method in which antibiotics are enclosed within the core of nanomaterials and is commonly used in the preparation of polymeric nanoparticles and liposomes. For example, polymeric nanoparticles made from poly (lactic-co-glycolic acid) (PLGA) are typically synthesized using an emulsion-solvent evaporation or nanoprecipitation technique (Gonzalez Gomez et al., 2019). In the emulsion-solvent evaporation method, PLGA is dissolved in an organic solvent and mixed with an aqueous solution of the antibiotic to form an oil-in-water (O/W) emulsion through vigorous stirring or ultrasonication. As the organic solvent evaporates, PLGA solidifies within the aqueous phase, thereby entrapping the antibiotic and forming nanoparticles. This strategy offers effective protection for the drug and improves its stability, while also allowing for relatively high drug loading efficiency. Nonetheless, the preparation process is more complex and may affect the drug release profile (Wang et al., 2025).

Chemical conjugation refers to the formation of covalent bonds between antibiotics and surface-functionalized nanomaterials. Representative reactions include EDC/NHS-mediated amide coupling (generally retaining 80%–95% activity), glutaraldehyde Schiff base cross-linking (possible 20%–40% activity loss), and bioorthogonal click chemistry (CuAAC) for high specificity. Cleavable linkers (disulfide, hydrazone) can be introduced to preserve function and enable triggered release (Girardot and Girardot, 1996; Sionkowska et al., 2024). Functional groups, such as amines or carboxyls, are incorporated onto nanoparticle surfaces, and cross-linking agents are used to covalently attach antibiotics to the carriers (Gonzalez Gomez et al., 2019). Choice of chemistry balances conjugation stability with preservation of antibiotic active sites. This strategy establishes robust chemical linkages between the drug and the carrier, effectively preventing premature drug release and markedly improving drug retention and delivery stability.

### 3.3 Principles of nanomaterial-mediated antibiotic delivery

Nanomaterials exhibit unique physicochemical properties that distinguish them fundamentally from conventional bulk materials, largely owing to their size-dependent and surface-dominated effects (Yi et al., 2024). These attributes profoundly influence drug adsorption, release, and targeting efficiency (Biesold et al., 2021). At the nanoscale, quantum confinement leads to changes in electronic energy states, which in turn modulate the optical, electrical, and magnetic behaviors of the materials. In drug delivery applications, small-sized nanoparticles can more easily penetrate biological barriers such as capillary walls and cellular membranes, thereby facilitating enhanced cellular uptake of therapeutic agents. Research indicates that nanoparticles with diameters smaller than 100 nm can enter cells via endocytosis, enabling intracellular drug delivery (Naruphontjirakul et al., 2018). Moreover, the surface properties of nanomaterials play a crucial role in drug delivery. Surface charge, hydrophilicity or hydrophobicity, and other physicochemical attributes can be precisely engineered through surface modification to achieve active targeting of specific cells or tissues. For instance, nanoparticles decorated with tumor-specific antibodies can selectively recognize and bind to cancer cells, thereby promoting preferential drug accumulation at tumor sites (Torrano et al., 2016; Maparu et al., 2022). Extending this principle to infectious/inflammatory niches, folate-decorated nanoparticles exploit overexpressed folate receptor-β (FRβ) on activated inflammatory macrophages, enhancing macrophage uptake and enabling localized delivery of antibiotics (e.g., vancomycin, rifampicin) within osteomyelitic lesions (Thomas et al., 2011). Likewise, anti-Staphylococcus aureus antibody-functionalized polymer nanoparticles exhibit selective adhesion to *S. aureus* biofilms and improve antibiotic concentration and biofilm disruption compared with non-targeted carriers, illustrating the utility of pathogen- or biofilm-specific immunoligands in augmenting local therapeutic efficacy (Aflakian et al., 2023).

Nanomaterial-mediated antibiotic delivery is a sophisticated and multifaceted process, the core of which lies in the precise encapsulation, transport, and targeted release of antibiotics at osteomyelitis infection sites (Hassani Besheli et al., 2017). Following encapsulation, nanocarriers can be administered either systemically or locally. To improve transport efficiency and stability *in vivo*, surface engineering strategies are frequently utilized (Cevc, 2004). Once the nanocarriers reach the infection site, targeted antibiotic delivery is accomplished through a combination of passive and active targeting mechanisms.

The advantages of nanomaterial-mediated antibiotic delivery are considerable. Conventional systemic antibiotic therapies often struggle to achieve therapeutic drug concentrations at osteomyelitic lesions and may result in unintended harm to healthy tissues. In contrast, nanocarriers can specifically direct antibiotics to infection site, enabling localized therapy, reducing systemic toxicity, and sparing adjacent healthy tissues from damage. In addition, nanocarriers provide controlled and sustained drug release, thereby overcoming the burst release issues associated with conventional carriers (Gounani et al., 2019; Birk et al., 2021). This capability allows for precise modulation of drug release kinetics and duration, ultimately improving therapeutic efficacy, minimizing drug wastage, and lowering overall treatment costs.

## 4 Delivery systems in osteomyelitis

**FIGURE 3:**
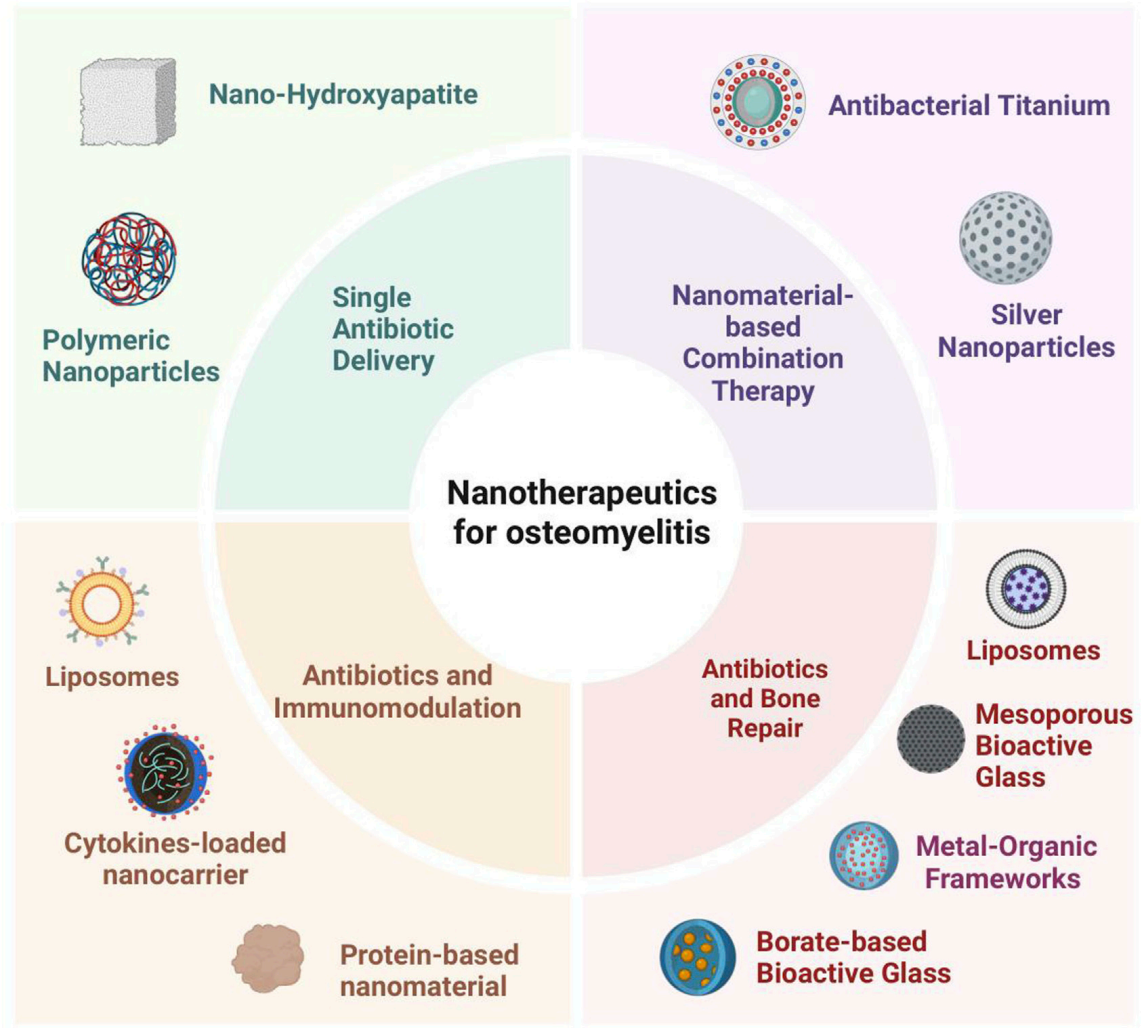
Application of nanodelivery systems in osteomyelitis: single antibiotic delivery, combination therapy, bone repair adjunct, and immunomodulation. Carriers include nHA, AgNPs, MOFs, liposomes, Ti, BBG, MBG, PNPs, protein- and cytokine-based systems.

This section focuses on functional strategies for the treatment of osteomyelitis–single antibiotic delivery, synergistic/multimodal therapy, immunomodulation, and bone repair. For each functional strategy, we summarize the most commonly applied material categories, compare their performance, and highlight conversion potential. The nano-delivery system has been applied in various ways in osteomyelitis, mainly through a single delivery method and synergistic delivery to regulate the immune system and promote bone repair, as shown in Figure 3.

### 4.1 Single antibiotic delivery

Conventional antibiotic delivery methods, such as oral and intravenous administration, face significant challenges in overcoming the physiological barriers of bone tissue during osteomyelitis treatment. Research indicates that, following traditional administration, antibiotic concentrations in bone tissue reach only 20%–30% of those in the bloodstream, which is insufficient to effectively eradicate bacteria embedded within biofilms. Moreover, frequent dosing increases the risk of drug accumulation in the body, potentially leading to adverse effects such as hepatic and renal toxicity (Thabit et al., 2019).

Degradable polymer carriers excel in the delivery of single antibiotics. Nanoparticles prepared from polylactic acid-hydroxyacetic acid copolymer (PLGA) can encapsulate antibiotics to achieve slow release lasting for weeks, such as vancomycin encapsulated in PLGA nanoparticles, which significantly increased the drug concentration at the site of infection and reduced systemic toxicity in animal experiments (Zirak et al., 2022).

Studies have shown that nano-antibiotic carrier systems are highly effective at suppressing bacterial proliferation. For example, Mohd Zaffarin et al. (2021), Padrão et al. (2021) a nanocarrier platform constructed by incorporating vancomycin into nanohydroxyapatite (nHA) exhibited pronounced antibacterial activity against major osteomyelitis pathogens, including *S. aureus* and *E. coli*.

Cytotoxicity assays serve as critical metrics for evaluating the safety of nano-antibiotic carrier systems. Evidence from various studies indicates that many nanomaterials exhibit excellent biocompatibility and minimal cytotoxicity. For example, copper-doped MBG nanoparticles loaded with antibiotics have been shown to exert negligible effects on the viability of normal cells, including fibroblasts and osteoblasts (Wang et al., 2016).

Comparative pharmacokinetic data show that nano/local systems markedly improve bone exposure versus free systemic drug: free vancomycin reaches only ≈20–30% of plasma levels in bone and falls below MIC within 6–8 h, whereas PLGA nanoparticles raise bone Cmax ≈2–3-fold and extend t1/2 to >24 h (Pisani et al., 2024); thermosensitive PLGA-PEG-PLGA gels maintain local concentrations >4 × MIC for 5–7 days (Yuan et al., 2022); PMMA or mesoporous silica-PMMA composites give an initial high local burst (>1,000 μg/mL eluate, first 24 h) followed by ≥ 10–20 × MIC for 1–3 weeks with low plasma levels; nHA or liposomal gentamicin similarly increases bone AUC (≈1.5–2×) while reducing renal accumulation (Rocha et al., 2022). These profiles collectively improve bone/plasma ratios, sustain antibacterial levels in biofilm niches, and lower systemic toxicity.

### 4.2 Nanomaterial synergistic therapy

Antimicrobial titanium materials achieve bactericidal functionality in titanium alloys via advanced surface modification techniques. Incorporating silver ions onto the titanium surface enables their gradual release, thereby suppressing bacterial proliferation. Combining antibiotics with antimicrobial titanium provides a dual antimicrobial effect. For example, Akshaya et al. (2022) coating the surface of titanium implants with antibiotic-loaded coatings effectively reduces the incidence of implant-associated osteomyelitis by both utilizing the mechanical properties of titanium to support bone tissue and by synergistically sterilizing the bone with antibiotics and silver ions.

Photothermal/photoacoustic nanoplatforms (e.g., polydopamine or black phosphorus coatings with vancomycin) reach 50 °C–55 °C under 808 nm irradiation, disrupt biofilm matrix, achieve additional 2-3 log10 CFU reductions versus antibiotic alone, and permit imaging-guided, on-demand release using gold nanorods or carbon dots, while avoiding collateral thermal damage (Mammari and Duval, 2023).

Several clinical trials investigating nanomaterial-mediated antibiotic delivery for osteomyelitis treatment are currently in progress. One research group assessed the efficacy of drug-loaded microporous nanohydroxyapatite beads in managing chronic osteomyelitis. In their study, vancomycin, gentamicin, and ceftriaxone were incorporated into the microporous nanohydroxyapatite beads at a 1:1:1 ratio to fill bone defects. This localized delivery approach proved effective in controlling infection and maintaining elevated local antibiotic concentrations for one to 6 weeks postoperatively (Jiamton et al., 2023). Evidence suggests that composite multifunctional antibiotic carriers based on nanoparticles can reduce or even eliminate the need for prolonged, repeated, and systemic antibiotic administration, as well as minimize or obviate surgical debridement of necrotic tissue (Uskokovic, 2015).

To facilitate antibiotic release, a novel micro-composite implant has been engineered for osteomyelitis management following total joint replacement. This approach involves incorporating drug-loaded mesoporous silica nanoparticles into polymethylmethacrylate (PMMA) bone cement, thereby enabling controlled antibiotic release. Such implants are capable of effectively eradicating infections and reducing the emergence of new drug-resistant bacterial strains, while preserving the spatial integrity and mechanical strength essential for joint fixation (Inceoglu et al., 2021). Similarly, PMMA bone cement is the clinical gold standard biomaterial for localized antibiotic therapy in osteomyelitis, and there are studies for slow and slow release of antibiotics and the release kinetics of antibiotics (Rajendran et al., 2021). Antibiotic-loaded PMMA also reduces the incidence of osteomyelitis infections and can be a useful option for long-term pharmacologic treatment of osteomyelitis (Wentao et al., 2017).

In order to address the side effects and resistance of antibiotics and to explore some local drug delivery systems for the treatment of osteomyelitis, some studies have prepared PLGA-PEG-PLGA copolymers, which are capable of slow-releasing antibiotics and promoting bone tissue repair, and have a wide range of potential for clinical applications (Yuan et al., 2022).

Immunomodulatory synergy further enhances outcomes: vancomycin-loaded mesoporous bioactive glass releasing Si/Ca ions shifts macrophages toward M2, lowers TNF-α/IL-6, and elevates osteogenic markers (ALP, RUNX2); antibiotic + IL-4 microsphere or folate-targeted M2-polarizing systems add ∼1–1.5 log10 extra CFU reduction and improve bone volume fraction compared with antibiotic alone (Rahaman et al., 2023).

### 4.3 Antibiotics and immunomodulation

The development of osteomyelitis is intimately linked to the host immune response. The inflammatory response triggered by bacterial infection activates the immune system, but an excessive immune response can also lead to bone tissue damage, while the presence of a bacterial biofilm evades recognition and clearance by the immune system, making the infection difficult to control (Wu S. et al., 2022). The pathogenesis of osteomyelitis is complex, and bacterial invasion of bone triggers an immune response in the body. Macrophages, as important members of the immune system, play a key role in the osteomyelitis process. Consequently, modulating the immune response is essential for effective osteomyelitis treatment.

The combination of immunomodulators and antibiotics offers a promising strategy to regulate immune homeostasis and enhance antimicrobial efficacy. For example, interferon-γ can enhance the phagocytosis of macrophages and promote their bacterial killing effect, which can improve the therapeutic effect when used in combination with antibiotics (Pomposello et al., 2020). In addition, anti-inflammatory cytokines such as interleukin-10 can mitigate excessive inflammatory responses and minimize bone tissue damage, demonstrating therapeutic potential for both bone regeneration and bone repair (Chen et al., 2025).

Nanomaterial-loaded antibiotics for the treatment of osteomyelitis offer distinct advantages. Nanomaterials can enhance the accumulation of antibiotics at infection sites, enable sustained and controlled drug release, maintain effective local drug concentrations, and thereby improve antimicrobial efficacy. Additionally, nanomaterials themselves can participate in immune modulation, influencing macrophage polarization. For example, nHA particles are capable of M2-type polarization of macrophages (Mahon et al., 2020). M2 macrophages secrete anti-inflammatory cytokines, such as IL-10, which help attenuate inflammation and facilitate tissue repair. The successful repair of bone defects by this functionalized scaffold in a rat femoral defect model confirms the role of nanomaterials in improving the local immune microenvironment of osteomyelitis and promoting bone healing by modulating macrophage polarization.

In terms of immunomodulation, nanomaterial-loaded antibiotics can also influence the crosstalk between osteoblasts and immune cells. For instance, one study developed a nanodrug delivery system incorporating silicon ions, in which MBG was coated onto nanohydroxyapatite carriers and loaded with vancomycin. In a chronic osteomyelitis animal model, this system not only effectively inhibited infection, but the presence of silicon ions also attenuated the cytotoxic side effects of vancomycin and enhanced bone regeneration at the infection site. These findings suggest that nanomaterial-based antibiotic delivery systems can modulate the immune microenvironment, thereby creating favorable conditions for osteoblast function and promoting bone tissue repair (Xu et al., 2021).

Nanomaterial-loaded antibiotics can positively affect the immune regulation by regulating macrophage polarization and influencing the interaction between osteoblasts and immune cells in the treatment of osteomyelitis, which opens up a new path for osteomyelitis treatment, and is expected to achieve better therapeutic effects in clinical applications.

### 4.4 Antibiotics and bone repair

The treatment of osteomyelitis requires addressing two major challenges simultaneously: effective infection control and bone repair. Traditional therapies often struggle to balance both aspects, leading to prolonged treatment durations and suboptimal prognoses (Ismat et al., 2021). Recently, integrated strategies combining antibiotics with bone regeneration have become a research focus, overcoming the limitations of conventional approaches. By leveraging innovative materials and technologies, these strategies seamlessly unite infection control with bone tissue regeneration, offering new perspectives and hope for the management of osteomyelitis and other complex bone diseases. This integrated approach is poised to transform the landscape of bone disease treatment.

In terms of infection control, the nano-antibiotic carrier system has demonstrated strong bactericidal ability. For example, borate glass has been developed as a local drug delivery vehicle for osteomyelitis treatment. In a rabbit tibial chronic osteomyelitis model, the ions released from borate glass were shown to support the proliferation, differentiation, osteogenic gene expression, and protein secretion of human bone marrow mesenchymal stem cells *in vitro*. Furthermore, after implantation into a rabbit tibia osteomyelitis model, the infection was effectively eradicated, and significant bone regeneration was observed after 12 weeks Nanomaterial-mediated antibiotic delivery (Egawa et al., 2020). This illustrates the dual functionality of such systems, providing both robust infection control and enhanced bone repair.

Nanomaterial-mediated delivery of antibiotics also promotes bone repair. At the cellular/molecular level, antibiotic-loaded nanoscaffolds increase early ALP activity, mineralized nodule formation, and upregulate RUNX2, COL1A1, OPN, OCN; ion-releasing (Si/Ca/B/Cu) platforms additionally elevate VEGF, linking angiogenesis with bone regeneration (Dousti et al., 2024; Lin et al., 2024).

For instance, one study developed an antibiotic-eluting collagen-hydroxyapatite scaffold, forming a layered dual-release system that could both eliminate infection and facilitate bone healing. In a mouse osteomyelitis model, vancomycin-eluting scaffolds significantly reduced *S. aureus* colonization in the tibia. Similarly, in a rabbit model of chronic osteomyelitis, these scaffolds promoted the healing of radial bone defects while eradicating *S. aureus* infection (Sheehy et al., 2025). In addition, graphene nanoparticles combined with mesenchymal stem cells have been shown to enhance cell differentiation, stimulate active bone formation, increase mineralization, and improve the repair of tibial bone defects in rats. These findings underscore the potential of nanomaterial-based delivery systems to integrate infection control with effective bone regeneration (Elkhenany et al., 2017).

There are also studies using liposome-encapsulated antibiotics to treat rabbit osteomyelitis models (Kadry et al., 2004). The results showed that co-treatment of bone tissue in the form of liposomes completely sterilized the bone tissue, and the liposomal formulation showed a lower incidence of nephrotoxicity and severe diarrhea than free drug treatment. There have also been studies using silver nanoparticles to treat a model of osteomyelitis in the tibia of rats and found that silver nanoparticles were effective against the pathogen, but that the use of nanosilver was close to the effectiveness of osteomyelitis treatment with a combination of classical antibiotics and local surgical debridement (Kemah et al., 2018).

Bioactive glass has natural antimicrobial properties and can inhibit the growth of osteomyelitis-causing bacteria even without the addition of antibiotics, in addition, drug-carrying bioactive glass showed good antimicrobial effects and bone regeneration in animal models (Zapata et al., 2022).

The effect of nano-antibiotic carrier system on osteoblast differentiation is also one of the focuses of research, certain nanomaterials can not only load antibiotics, but also promote the differentiation and proliferation of osteoblasts, for example, Xu et al. (2021) the nano-loading system constructed by wrapping MBG containing silica ions around nHA-loaded vancomycin can significantly increase the activity of alkaline phosphatase (ALP) in osteoblasts, and promote the calcium nodule Formation. Collectively, a cascade emerges: localized antibiotic release clears infection, attenuates excessive inflammation, re-polarizes macrophages, and permits sustained activation of osteogenic programs culminating in accelerated matrix mineralization and structural bone regeneration (Gou et al., 2024).

## 5 Challenges and prospects

Nanomaterial-mediated antibiotic delivery has brought about revolutionary changes in the treatment of osteomyelitis, demonstrating significant research significance and enormous application potential. Common nanomaterials for antibiotic delivery are shown in Table 2. Nanomaterial-mediated antibiotic delivery for osteomyelitis treatment faces many challenges and solutions.

**TABLE 2:** Overview of applications of nanomaterials in osteomyelitis treatment.

Type of nanomaterial	Delivery system	Mechanism of action	Therapeutic effect	Advantages	Limitations	Discussed in next	Reference
Nano-hydroxyapatite (nHA)	Drug-loaded microspheres/bone scaffolds	Provides bone-like scaffold, promotes osteoblast adhesion and differentiation, enables sustained antibiotic release	Antibacterial, promotes repair of bone defects in chronic osteomyelitis	Excellent biocompatibility and osteoconductivity	Limited antibacterial effect alone, often requires combination with drugs, complex preparation	3.1; 4.1; 4.3; 4.4	Safi et al. (2018)
Mesoporous Bioactive Glass (MBG)	Drug-loaded bioactive bone fillers/scaffolds	High specific surface area for drug loading, releases bioactive ions to promote osteogenesis and inhibit osteoclasts	Inhibits infection and promotes new bone formation *in vitro* and *in vivo*	High drug loading capacity, promotes bone regeneration via ion release	Complex preparation, pH changes during degradation need to be controlled	3.1; 4.1; 4.3; 4.4	Sui et al. (2018)
Polymeric Nanoparticles	Biodegradable nanocarriers	Encapsulates antibiotics for controlled degradation and sustained release	Increases local drug concentration, reduces systemic toxicity	Flexible design, controlled drug release, high drug loading capacity	Complex preparation, risk of initial burst release	3.1; 3.2	Dhas et al. (2018)
Aragonite Nanoparticles	Local antibiotic nanocarriers	Delivers antibiotics locally with controlled release	Effective antimicrobial activity, promotes biocompatibility	Natural origin, bioresorbable and biocompatible	Long-term metabolic safety not fully studied	3.1	Papa et al. (2009)
Liposomes	Lipid-based vesicles	Encapsulates hydrophilic/hydrophobic drugs, enables cell uptake via fusion or endocytosis	Clears infected bone, reduces nephrotoxicity	High biocompatibility, protects drug stability, modifiable surface	Poor physicochemical stability *in vivo*, high cost	3.1; 3.1; 4.4	Navarro-Partida et al. (2021)
Metal-Organic Frameworks	Porous nanocarriers	Adsorbs antibiotics, enables controlled release via pH-responsive degradation	Sustained drug release at infection sites, potential for immune modulation	Large specific surface area, modular design, multifunctional potential	Need to assess *in vivo* safety, biodegradability, and ion toxicity	3.1	Kang et al. (2025)
Antibacterial Titanium (Ag-coated)	Surface-functionalized implants	Slowly releases silver ions from titanium surface, provides mechanical support simultaneously	Reduces implant-associated infections, enhances osseointegration	Strong synergy between structural support and antibacterial effect	Issues with release kinetics and long-term safety of silver ions, high cost	3.1	Piotrowska et al. (2017)
Silver Nanoparticles	Antibacterial agents	Disrupts bacterial membranes, proteins, and DNA	Effective against pathogens, comparable to antibiotics combined with debridement	Broad-spectrum bactericidal effect, synergistic with antibiotics	Limited efficacy when used alone, potential toxicity	3.1; 4.4	Luceri et al. (2023)
Borate Bioactive Glass	Drug-loaded scaffolds/implants	Releases ions to support stem cell differentiation and bone matrix secretion	Cures chronic osteomyelitis	Natural antibacterial property, promotes osteogenesis	Fast degradation rate, stability needs to be controlled	3.1	Ensoylu et al. (2022)
Graphene Nanoparticles	Stem cell delivery platforms	Enhances osteogenesis of mesenchymal stem cells, serves as a cell adhesion platform	Promotes mineralization and healing of bone defects	Osteoinductive and osteoconductive	Potential risk of long-term inflammation or toxicity	4.4	Slita et al. (2018)
Mesoporous Silica Nanoparticles	Drug delivery particles/cement additives	Adsorbs antibiotics via electrostatic or ligand interactions, high surface functionality	Controls infection, maintains mechanical strength in PMMA	Easy functionalization, stable chemical properties	Limited binding strength, risk of premature drug leakage	3.2; 4.2	Wan et al. (2024)

The safety of nanomaterials is a primary concern, including potential risks in terms of cytotoxicity, immunogenicity, and long-term *in vivo* metabolism, which require in-depth studies to ensure their safety for human applications (Lee et al., 2019; Lu and Fan, 2024; Wang et al., 2024). Current toxicological evaluation incorporates: (1) acute/sub-chronic toxicity (MTD, NOAEL) in rodent models (Guo et al., 2022); (2) organ biodistribution/accumulation via ICP-MS or fluorescence (liver-spleen transient uptake with PLGA or black phosphorus clearing within 14–28 days; persistent silver >10 mg/kg may induce mitochondrial swelling) (Mohammadpour et al., 2019); (3) histopathology (H&E), serum biochemistry (ALT, AST, BUN, creatinine), hematology, hemolysis, complement activation (CH50), cytokine panels (IL-6, TNF-α, IL-1β), macrophage polarization profiling (CD86/CD206), genotoxicity (Ames, micronucleus), and immunogenicity (anti-carrier antibody ELISA); representative vancomycin–PLGA or nHA systems show no significant elevation of hepatic/renal markers and reversible mild splenic macrophage loading (Parisi et al., 2018).

Preparation process and cost issues are also important factors limiting their wide application. Currently, the large-scale preparation technology of nanomaterials is still not perfect and the cost is high, which hinders their clinical promotion (Zhang X. et al., 2023). Mainstream scalable fabrication routes include nanoprecipitation, emulsification-solvent evaporation, spray drying, microfluidic continuous flow (parallelized chips), supercritical fluid processing, melt extrusion (polymer-drug solid dispersions), sol-gel (bioactive glass), layer-by-layer assembly, and 3D printing of composite scaffolds; approximate pilot costs: PLGA or lipid nanoparticles ≈ USD 5–20/g (carrier) excluding API(Kumar et al., 2023), mesoporous silica ≈ USD 10–30/g, while functional (multi-responsive) constructs can exceed USD 50/g (Tenchov et al., 2025); key scale-up bottlenecks involve batch reproducibility (size/PDI drift), solvent residual compliance (ICH Q3C), sterilization-induced degradation (γ, e-beam), lyophilization stability (cryoprotectant selection), continuous in-line PAT (NIR/DLS analytics), and regulatory CMC definition of complex multi-component systems.

In addition, the effect of nanomaterial-mediated antibiotic delivery on antibiotic resistance is still unclear, and further research is needed to avoid or reduce the emergence of resistance (Wang and Vikesland, 2023). While high local concentrations typically suppress resistance selection, potential new mechanisms include: (a) sub-MIC “tail” release promoting tolerance/persister enrichment; (b) biofilm penetration gradients inducing efflux pump upregulation; (c) co-delivered metal ions (Ag, Cu) co-selecting metal-antibiotic resistance genes; (d) surface-adaptive mutations to persistent nano-bio interfaces; mitigation strategies involve PK/PD modeling to eliminate sub-MIC tails, combinational stimuli (photothermal + antibiotic), periodic triggerable pulses, and longitudinal metagenomic surveillance (mecA, van, bla, efflux operons) over serial passages—early studies show slower MIC shift trajectories versus free-drug regimens when burst + sustained profiles are optimized (Soto, 2013; Hajiagha and Kafil, 2023).

In the future, the research on nanomaterial-mediated antibiotic delivery for osteomyelitis will primarily focus on the development of intelligent nanocarriers, the integration of multimodal therapeutic strategies, and advancing clinical translation (Sadowska et al., 2021; Zarepour et al., 2024; Nguyen Ngoc et al., 2025). The design of intelligent nanocarriers aims to achieve precise regulation and active targeting of drug release, thereby enhancing therapeutic efficacy (Agiba et al., 2024). Representative “smart” systems include pH-responsive ZIF-8/polymer hybrids releasing cargo in acidic infection niches (pH ≈ 6.5), enzyme-responsive gelatin/MMP- or bacterial β-lactamase-cleavable linkers for on-demand gentamicin or vancomycin liberation, ROS-triggered thioketal or boronic ester platforms accelerating release under oxidative bursts, NIR photothermal/photodynamic dual-responsive black phosphorus or polydopamine composites, magnetic or ultrasound-triggered phase-change liposomes enabling spatiotemporal pulses, and multi-responsive injectable hydrogels (recent patents integrating pH + enzyme + photothermal modules) that couple theranostic imaging (photoacoustic/fluorescence) with controlled antibiotic dosing (Gade et al., 2024; Su et al., 2025; Zhang R. et al., 2025). Moreover, combining nanomaterial-based antibiotic delivery with multimodal therapies—such as photothermal therapy, gene therapy, and immunotherapy—can exert synergistic effects, further improving treatment outcomes and reducing the risk of drug resistance (Mammari and Duval, 2023; Sharma et al., 2024). Clinical translation will depend on harmonized characterization standards (size, surface chemistry, release kinetics), validated immunotoxicity panels, accelerated stability protocols, cost-reduction via continuous manufacturing, and early phase adaptive trial designs capturing both infection eradication and bone regeneration endpoints. In parallel, intensified efforts in clinical translational research will address issues related to the safety, quality control, and cost of nanomaterials, facilitating the transition of these technologies from laboratory research to clinical application. Ultimately, these advancements are expected to provide more effective therapeutic options for patients with osteomyelitis (Csóka et al., 2021). Scalable production requires processes that ensure batch-to-batch reproducibility, sterility, and cost-effectiveness. Techniques operating at benchtop scale must be validated for use in large reactors, with attention to solvent removal, residues, and downstream purification, and economic analysis should be considered early to assess the feasibility of widespread clinical deployment.

Nanomaterial-mediated delivery of antibiotics brings new hope for the treatment of osteomyelitis. Despite the challenges, with the deepening of research and the continuous progress of technology, it is expected to make greater breakthroughs in the field of osteomyelitis treatment, improve the quality of life of patients, and reduce the burden on society.

## 6 Conclusion

Nanomaterial-mediated antibiotic delivery has fundamentally transformed the treatment landscape for osteomyelitis, demonstrating significant research value and tremendous application potential. The traditional treatment of osteomyelitis has many limitations, such as large surgical trauma, numerous side effects of systemic antibiotic therapy, and poor local drug delivery effects. Nanomaterials, owing to their unique physical and chemical properties, offer innovative solutions to these challenges.

The antibiotic delivery strategy mediated by nanomaterials, leveraging its unique physical-chemical characteristics and biological activities, has opened up a brand-new path for osteomyelitis treatment. In terms of therapeutic advantages, nanocarriers can achieve local targeted delivery and controlled release of antibiotics. This not only significantly increases the drug concentration at the infection site, effectively inhibiting bacterial growth and inflammatory responses, but also reduces side effects caused by systemic medication. Meanwhile, the material properties can promote bone tissue repair and regeneration.

Although challenges remain, nanomaterial-mediated antibiotic delivery brings renewed hope to osteomyelitis management. With ongoing research and technological progress, this approach is expected to achieve even greater breakthroughs, improving patient outcomes and quality of life while reducing the societal burden of the disease. Future research priorities: targeted ligands; standardized safety frameworks; PK/PD-guided smart multi-responsive delivery; robust large-animal and adaptive clinical studies with dual endpoints; staged combinational (antimicrobial + immune + pro-angiogenic) strategies; scalable continuous manufacturing plus open data and long-term host/microbiome monitoring.
